# Composition-Dependent Cytotoxic and Antibacterial Activity of Biopolymer-Capped Ag/Au Bimetallic Nanoparticles against Melanoma and Multidrug-Resistant Pathogens

**DOI:** 10.3390/nano12050779

**Published:** 2022-02-25

**Authors:** Alfonso Nieto-Argüello, David Medina-Cruz, Yeremi S. Pérez-Ramírez, Sergio A. Pérez-García, Miguel A. Velasco-Soto, Zeinab Jafari, Israel De Leon, María Ujué González, Yves Huttel, Lidia Martínez, Álvaro Mayoral, Thomas J. Webster, José M. García-Martín, Jorge L. Cholula-Díaz

**Affiliations:** 1 School of Engineering and Sciences, Tecnologico de Monterrey, Eugenio Garza Sada 2501, Monterrey 64849, NL, Mexico; alfonsonieto25@gmail.com (A.N.-A.); yeremi_said@hotmail.com (Y.S.P.-R.); miguel.velasco85@tec.mx (M.A.V.-S.); zjafari@tec.mx (Z.J.); ideleon@tec.mx (I.D.L.); 2 Department of Chemical Engineering, Northeastern University, Boston, MA 02115, USA; davidmedinacrz@gmail.com (D.M.-C.); websterthomas02@gmail.com (T.J.W.); 3 Centro de Investigación en Materiales Avanzados, S. C. (CIMAV), Unidad Monterrey, Alianza Norte 202, Apodaca 66628, NL, Mexico; alfonso.perez@cimav.edu.mx; 4 Instituto de Micro y Nanotecnología, IMN-CNM, CSIC (CEI UAM+CSIC), Isaac Newton 8, 28760 Tres Cantos, Spain; maria-ujue.gonzalez@csic.es (M.U.G.); josemiguel.garcia.martin@csic.es (J.M.G.-M.); 5 Instituto de Ciencia de Materiales de Madrid, ICMM-CSIC, Sor Juana Inés de la Cruz 3, 28049 Madrid, Spain; huttel@icmm.csic.es (Y.H.); lidia.martinez@icmm.csic.es (L.M.); 6 Instituto de Nanociencia y Materiales de Aragón (INMA), CSIC-Universidad de Zaragoza, Pedro Cerbuna, 50009 Zaragoza, Spain; amayoral@unizar.es; 7 Laboratorio de Microscopías Avanzadas (LMA), Universidad de Zaragoza, 50018 Zaragoza, Spain; 8 Center for High-Resolution Electron Microscopy (CħEM), School of Physical Science and Technology (SPST), ShanghaiTech University, 393 Middle Huaxia Road, Pudong, Shanghai 201210, China

**Keywords:** nanomedicine, bimetallic nanoparticles, antimicrobial resistance, anticancer activity, green chemistry

## Abstract

Nanostructured silver (Ag) and gold (Au) are widely known to be potent biocidal and cytotoxic agents as well as biocompatible nanomaterials. It has been recently reported that combining both metals in a specific chemical composition causes a significant enhancement in their antibacterial activity against antibiotic-resistant bacterial strains, as well as in their anticancer effects, while preserving cytocompatibility properties. In this work, Ag/Au bimetallic nanoparticles over a complete atomic chemical composition range were prepared at 10 at% through a green, highly reproducible, and simple approach using starch as a unique reducing and capping agent. The noble metal nanosystems were thoroughly characterized by different analytical techniques, including UV-visible and FT-IR spectroscopies, XRD, TEM/EDS, XPS and ICP-MS. Moreover, absorption spectra simulations for representative colloidal Ag/Au-NP samples were conducted using FDTD modelling. The antibacterial properties of the bimetallic nanoparticles were determined against multidrug-resistant *Escherichia coli* and methicillin-resistant *Staphylococcus aureus*, showing a clear dose-dependent inhibition even at the lowest concentration tested (5 µg/mL). Cytocompatibility assays showed a medium range of toxicity at low and intermediate concentrations (5 and 10 µg/mL), while triggering an anticancer behavior, even at the lowest concentration tested, in a process involving reactive oxygen species production per the nanoparticle Au:Ag ratio. In this manner, this study provides promising evidence that the presently fabricated Ag/Au-NPs should be further studied for a wide range of antibacterial and anticancer applications.

## 1. Introduction

Cancer is one of the most concerning diseases of the present era. Data from the American Cancer Institute estimates that by 2021 approximately 1.9 million people will be diagnosed with cancer and, consequently, 600,000 will die [1]. Moreover, data from the National Cancer Institute (NIH) indicated that in 2018 alone, US expenses for cancer totaled 150.8 billion dollars [2], and both cancer prevalence and cost will rise over the next few years by approximately 30.2 million new cases by 2040 [3]. Undoubtedly, cancer studies have been at the forefront of medical research for years, however, improved efforts are still needed to develop novel and cost-effective materials to defeat such diseases. Besides this, current treatments are often ineffective, triggering the appearance of both life-compromising side effects and drug resistance by tumors [4]. For instance, some studies have shown that cancer cells exhibit resistance to cisplatin (a recurrent chemotherapy drug) in elevated numbers in patients with colorectal, lung, ovary and prostate cancer [5].

On the other hand, the healthcare system is also fighting a constant rise in antimicrobial resistance (AMR) to antibiotics which is now a worldwide crisis [6]. Data from the Centers for Disease Control and Prevention (CDC) now categorizes 18 bacterial strain-related diseases as urgent, serious and highly concerning [6]. This means that there are few available antibiotics to treat such bacteria. For instance, methicillin resistant Staphylococcus aureus (MRSA) caused approximately 320,000 infections in 2017 in the USA [7,8]. This trend is problematic as there are more than 2.8 million people diagnosed with AMR-related bacterial infections each year in the USA, with more than 35,000 associated deaths [6]. In addition, the economic cost from AMR in 2018 was estimated at 3 billion dollars [9]. Unsurprisingly, the number of new AMR cases is expected to rise in the years ahead. Indeed, the World Health Organization (WHO) has estimated that AMR will become the number one reason for death worldwide by 2050 [10,11], while the economic impact of AMR in the healthcare system will rise to 1 trillion dollars [12]. Moreover, cancer patients often suffer from AMR infections due to their weakened immune system.

Therefore, cancer and AMR are life-threatening concerns whose outcome is expected to decline leading to a clear need for identifying novel solutions. For such solutions, researchers have focused on the nanoscale, and indeed, nanotechnology may have the solution. The field of nanotechnology, understood as the scientific and technological discipline that employs materials less than 100 nm in at least one dimension [13], has grown in interest over the past few several decades as it is a powerful tool to solve problems pertinent for almost any industry. Nanomaterials (NMs) exhibit unique chemical, biological, optical, physical, magnetic and electric properties when compared to their bulk counterparts. The nanoworld has flourished when applied to medicine, leading to the birth of nanomedicine [14]. Different nanomaterials, such as metals, metalloids and polymers, have all shown promising antibacterial, anticancer and bioimaging properties without compromising the viability of healthy tissue [15,16].

Nevertheless, the use of nanoscale materials in medicine has some drawbacks, which indeed limit their application. The majority of NMs are currently produced by physicochemical approaches (e.g., laser ablation or chemical reduction), and their production is commonly linked to the production of toxic by-products, especially in the case of traditional chemical methods [17]. Besides this, some NM fabrication methods are costly and present a negative impact on the environment [18]. One of the most concerning limitations arises from the interaction between NMs and biological tissue, leading to cytotoxicity which can be attributed to almost any type of NM regardless of the synthesis method used. Moreover, most NMs produced by physicochemical methods require chemical functionalization after synthesis in order to promote biocompatibility, adding an extra synthetic step to achieve this behavior [19,20].

Consequently, there is an increasing need for environmentally friendly, cost-effective and safe NM synthesis methods. This has led to the creation of a new field termed green nanotechnology. Green nanotechnology is characterized by the use of living organisms, such as bacteria, fungi and mammalian cells, plant extracts or specific biomolecules (e.g., carbohydrates) and naturally-derived waste materials to produce NMs [21]. One important advantage of green synthesized NMs is related to the organic corona present after synthesis, composed of biomolecules from the raw materials used, which eliminates the need for extra functionalization and provides for excellent biocompatibility once the NMs are used in biological systems [22,23]. For instance, bacteria-mediated selenium nanoparticles (SeNPs) showed antibacterial activity against MRSA and multidrug-resistant *Escherichia coli* (MDR-EC) without inducing any significant cytotoxic effects against human dermal fibroblasts (HDF) [24]. The SeNPs possessed a protein corona derived from the bacteria, increasing structural biocompatibility [24]. In a more recent study, Srivastava et al. synthesized silver nanoparticles (AgNPs) using different biopolymers (alginate, gelatin and reconstituted silk fibroin) as capping agents [25]. They observed that biopolymer-capped AgNPs had a negligible haemolysis effect in vivo (Balb/c mice). Thus, green-synthesized NMs possess enhanced biomedical properties compared to their physiochemically-synthesized counterparts.

There have been attempts to produce bimetallic nanoparticles (BMNPs) following green chemistry approaches since they can combine the attractive properties of two metals (e.g., silver (Ag) and gold (Au)) generating a synergetic effect enhancing performance [26]. Both Ag and Au are preferable materials for BMNPs over other metals due to their reliability, established and cost-effective synthesis and intrinsic properties (such as cytotoxicity against cancer cells and antibacterial properties) [27]. Moreover, it has been demonstrated that the combination of Ag with noble metals (e.g., Au, Pt, Pd or Ir) promotes the oxidative dissolution of Ag as Ag ions (Ag+), enhancing antimicrobial and anticancer activity [28,29,30,31,32]. Therefore, Ag-based bimetallic NPs, such as Ag/Au-NPs, seem to be good candidates as improved antimicrobial and anticancer agents.

Although some applications of Ag/Au materials can be found in the literature, few efforts have focused on nanomedicine. As an example, Ag/Au-NPs were synthesized employing *Stigmaphyllon ovatum* (leaf extract) showing anticancer activity against human cervical carcinoma [33]. In addition, Gopinath et al. reported that Ag/Au-NPs synthesized with *Gloriosa superba* (leaf extract) possessed antibacterial and antibiofilm activity against *Bacillus subtilis* and *Escherichia coli* [34]. Meanwhile, Bankura et al. studied the antibacterial properties of Ag/Au-NPs synthesized using dextran against *Bacillus subtilis*, *Bacillus cereus*, *Escherichia coli* and *Pseudomonas aeruginosa* [35]. As noted, the above-described strategies are based on the use of plant extracts, however, one of the main drawbacks of these approaches is that the specific phytochemicals responsible for serving as the reducing and capping agents have not been well defined, which compromises the reproducibility of the synthesis method. In this regard, the use of a specific reactant as a dual agent, e.g., a biopolymer such as starch, would allow researchers to determine the specific nature and concentration of the reagents, making such green methodologies highly reproducible and reliable [36,37].

Further, despite the aforementioned advantages of BMNPs over monometallic NPs, there have been few reports concerning the application of bimetallic nanomaterials against clinically relevant multidrug-resistant microbial strains. For instance, Padilla-Cruz et al. recently reported the antibacterial properties of green-synthesized Ag–Fe BMNPs against multidrug-resistant *Staphylococcus aureus* and *Pseudomonas aeruginosa* [38]. Zhao et al. applied AuRh BMNPs as nano-antibiotics against MDR-EC, MDR *Klebsiella pneumoniae*, polymyxin-resistant (PR) *Escherichia coli* and PR *Pseudomonas aeruginosa*, showing minimal inhibitory concentrations (MIC) lower than 30 μg/mL for all of the MDR bacteria tested [39]. In our previous work, we reported the antibacterial activity of starch-capped AuNPs and Ag/Au-NPs at an atomic ratio of 50:50 against MRSA and MDR-EC [36]. The monometallic AuNPs were not toxic to either human dermal fibroblasts (HDF) or melanoma cells, while the Ag/Au-NPs were cytocompatible towards HDF cells, but a dose-dependent anticancer effect was found when human melanoma cells were exposed to the BMNPs [36]. In this context, the present study examined whether the chemical composition of Ag/Au-NPs improved antibacterial and anticancer properties compared to their monometallic counterparts and, thus, could be further explored for clinical applications.

Herein, Ag/Au bimetallic nanoparticles were synthesized using a reproducible and simple green method employing starch as a reducing and capping agent. The nanoparticles were characterized by UV-vis spectroscopy to determine their optical properties and stability. The optical properties of the nanomaterials were further studied through three-dimensional finite difference time domain (3D FDTD) simulations to verify the experimental results. NP properties were analyzed by Fourier Transform infrared (FTIR) spectroscopy and X-ray diffraction (XRD), whereas the morphology and size were evaluated by transmission electron microscopy (TEM), and the composition determined by energy-dispersive X-ray spectroscopy (EDX), X-ray photoelectron spectroscopy (XPS) and inductively coupled plasma mass spectrometry (ICP-MS). The antibacterial and anticancer activity of the Ag/Au-NPs was studied in vitro against MDR-EC and MRSA, and human melanoma cells, respectively. Lastly, reactive oxygen species (ROS) experiments were conducted to elucidate cell response mechanisms to the BMNPs.

## 2. Materials and Methods

### 2.1. Chemicals

Sodium tetrachloroaurate (III) dihydrate (NaAuCl_4_:2H_2_O; 99%) was purchased from Sigma-Aldrich (St. Louis, MO, USA), silver nitrate (AgNO_3_; 99.70%) was purchased from J.T. Baker (Mexico City, Mexico), and starch was purchased from CTR Scientific (Monterrey, NL, Mexico). The pH of the starting reaction solution was adjusted with sodium hydroxide (NaOH; 98.40%, CTR scientific, Monterrey, NL, Mexico). All of the experiments were carried out using deionized water and all the chemicals were used without further purification. Besides this, all of the glassware and stirring bars employed in all the synthetic steps were kept in an 11.11% hydrochloric acid (HCl; 37%, Sigma-Aldrich St. Louis, MO, USA) solution for 24 h, then in a potassium hydroxide/ethanol (KOH/EtOH; KOH ≥ 85%, Sigma-Aldrich St. Louis, MO, USA and EtOH reagent grade, CTR Scientific, Monterrey, NL, Mexico) solution during the same amount of time, and were finally rinsed with enough water prior to their use.

### 2.2. Colloidal Ag/Au-NPs Synthesis

Ag/Au-NPs were synthesized following a previously published method [36,37]. The synthesis procedure was as follows: an aqueous solution of 1% *w/v* starch was prepared and maintained under constant reflux and magnetic stirring for 30 min; subsequently, the solution was centrifuged for 10 min at 3500 rpm. The supernatant of the starch solution was added to DI water previously heated to 70 ± 1 °C with a pH of 11.11 ± 0.10 achieved by the addition of NaOH. Afterwards, the precursors in solution form (dependent on the desired composition) were added. Table S1 in the Supplementary Information section summarizes the experimental parameters of the reagents used for the synthesis of the Ag/Au-NP colloids. The reaction occurred in a magnetic stirrer for 3 h in a water bath at a constant temperature of 70 ± 1 °C. The synthesized colloidal Ag/Au-NPs were centrifuged twice for one hour at 12,500 rpm. The supernatants were discarded, and the final pellet was dispersed in 10 mL of DI water. The final colloids were further characterized and used for in vitro analysis as described below.

### 2.3. Characterization Techniques

#### 2.3.1. UV-Visible Spectroscopy

The optical properties of the colloidal Ag/Au-NP samples were examined using a PerkinElmer 365 spectrometer (PerkinElmer Inc. Waltham, MA, USA) in a range from 300 to 800 nm with a speed scan of 20 nm/min and a scan step of 0.5 nm. The colloidal NPs were diluted at a 1:5 ratio with DI water to measure their absorption spectra. The absorption spectra of the Ag/Au-NPs were measured on the same day of their syntheses (0 day) and on subsequent days (up to 43 days) to demonstrate the stability of the colloids. Likewise, the spectra from the different batches were compared to assess reproducibility.

#### 2.3.2. FDTD Modelling

Absorption spectra simulations for representative colloidal Ag/Au-NP samples were carried out using a 3D FDTD solver (Lumerical). An isolated spherical nanoparticle with a diameter of 12.5 nm and immersed in water was used for all of the simulations. Using a constant diameter for all of the bimetallic nanosystems in our simulations was a valid approximation because the size of the synthesized nanoparticles is much smaller than the range of wavelengths used in the model. This implies that the resonance wavelength depends only on the optical constant of the alloys [40], which were taken from the experimental data reported by Peña-Rodríguez et al. [41]. For the simulations of pure Au and Ag, we used previously reported optical constants [42,43]. To ensure accuracy, the minimum mesh size was set to 0.2 nm, and perfectly matched layers were used in all boundaries limiting the numerical space. The absorbed power was calculated as the difference between the incident power and the power exiting a closed volume surrounding the nanoparticle.

#### 2.3.3. X-ray Diffraction Analysis

Powder XRD diffraction patterns for each of the mono- and bimetallic Ag/Au-NPs were acquired on a Rigaku MiniFlex 600 diffractometer operating at a voltage of 40 kV, a current of 15 mA, and with Cu-Kα radiation (λ = 1.542 Å). All patterns were collected at room temperature with a step width of 0.05° (2θ) and a scan speed of 0.2°/min. The sample preparation consisted of drying dropwise 8 mL of the colloidal metal NPs on a cover glass used as sample holder at 50 °C.

#### 2.3.4. Fourier Transform Infrared Spectroscopy

FTIR spectra were taken using a PerkinElmer Spectrum 400 FT-IR/FT-NIR (Perkin-Elmer, Waltham, MA, USA) in attenuated total reflectance (ATR) mode. The IR spectra were obtained by scanning at a range of 380 to 4000 cm^−1^ and with a resolution of 4 cm^−1^. SpectrumTM software from Perkin-Elmer was used to normalize and correct the baseline for all of the IR spectra. For FTIR spectroscopy measurements, 5 μg of the dried noble metal NP sample from XRD analysis was used.

#### 2.3.5. Inductively Coupled Plasma Mass Spectrometry

The chemical composition of the colloidal mono- and bimetallic NPs was determined by ICP–MS. The measurements were performed in a Thermo Scientific iCAP 6500-ICP-OES CID spectrometer (Thermo Scientific, Waltham, MA, USA), using a wavelength of λ = 328.1 and 242.8 nm for Ag and Au, respectively. The reference samples used included a 1000 μL/mL Ag solution, a 1000 μL/mL Au solution and a 100 μg/mL Au solution each in 2% HNO_3_ from AccuStandard. Analytical quality control for the quantification of silver and gold was asserted by multiple blanks and calibration curves using the reference samples, thereby acquiring a correlation coefficient of 0.99997 and 0.99995, respectively. The method of detection limits were 0.12 and 2.0 μg/mL for Ag and Au, respectively.

#### 2.3.6. X-ray Photoelectron Spectroscopy

XPS measurements were conducted using an Escalab 250Xi XPS spectrometer with a monochromatic Al K-α source (1486.6 eV), spot size of 650 µm, base pressure of 10^−10^ mbar, energy pass of 40 eV with a 0.5 eV step size for survey scans, 20 eV pass energy and 0.1 eV step size for core level acquisition. Calibration was performed by adjusting the C1s signal at 285 eV for all samples. The X-ray voltage and power were 14 kV and 350 W, respectively. Samples were measured without any further treatment, consisting of a dried drop casted onto a copper substrate, which was previously cleaned with sandpaper and sonicated with isopropanol and used immediately. Concentration analyses were conducted with Thermo Avantage v5.9915 Surface Analysis Software extracted from wide scan spectra areas and considering the sensitivity factors for each XPS peak.

#### 2.3.7. Scanning Transmission Electron Microscopy

The Ag/Au-NPs were characterized by means of spherical aberration corrected scanning transmission electron microscopy (Cs-corrected STEM) using an annular dark field detector (HAADF). STEM inspection was performed using a FEI Titan XFEG transmission electron microscope equipped with a CEOS spherical aberration corrector for the electron probe. Prior to the experiments, aberrations were corrected using a gold standard sample assuring a spatial resolution of 0.8 Å. For chemical analyses, the microscope was also fitted with a Gatan Tridiem energy filter (GIF) and with a silicon drift detector (SDD) Oxford energy dispersive X-ray (EDX) spectrometer. Chemical information was acquired by means of EDX spectroscopy. For electron microscopy STEM inspection, the colloidal Ag/Au-NPs were first sonicated to minimize particle agglomeration, and then a few drops of the suspension were deposited onto holey carbon copper microgrids.

### 2.4. Biomedical Properties Study

#### 2.4.1. In Vitro Antimicrobial Studies

For the in vitro antimicrobial studies, strains of Gram-positive and -negative bacteria were used: MRSA (ATCC 4330; ATCC, Manassas, VA, USA) and MDR *E. coli* (ATCC BAA-2471; ATCC, Manassas, VA, USA), respectively. Briefly, bacterial cultures were inoculated in Luria-Bertani (LB) (bioPLUS, bioWORLD) medium and incubated at 37 °C and 200 rpm for 24 h. The optical density (OD) of the bacterial cultures was measured at 600 nm using a spectrophotometer (SpectraMax M3, Molecular Devices, Sunnyvale, CA, USA), after which bacterial suspensions were diluted to a concentration of 10^6^ colony-forming units per milliliter (CFU/mL). Growth curves and standardization of correlation between OD and CFU/mL were completed for each bacterium in media prior to the completion of both the antibacterial growth curve analysis and colony counting unit assays.

For the growth curve antimicrobial assay, 100 μL of 4 different Ag/Au-NPs (20:80, 40:60, 60:40 and 80:20) were added to the LB medium at a set range of concentrations and mixed with 100 μL of bacteria in LB medium. The mixtures were then added to a 96-well plate (Thermo Fisher Scientific, Waltham, MA, USA) to a final volume of 200 μL. For the untreated controls, 100 μL of bacteria were mixed with 100 μL of LB medium without BMNPs. Once the plate was prepared, the absorbance of all samples was measured at 600 nm on an absorbance plate reader every 2 min for 24 h with no shaking. Negative controls containing only BMNPs and media were used to determine the absorbance caused by the BMNPs.

For the colony counting assays, bacteria were seeded in a 96-well plate and treated with different concentrations of the BMNPs for 8 h inside an incubator at 37 °C. Then, the 96-well plate was removed from the incubator, and all the samples were diluted with phosphate buffer saline (PBS) in a series of vials to either ×100, ×1000, or ×10,000. 10 µL aliquots of each dilution were then dropped on the Agar plates, and were then incubated for 8 h inside the incubator at 37 °C. The resulting number of colonies formed in each plate was counted at the end of the incubation time.

#### 2.4.2. Determination of the Minimum Inhibitory Concentration

As an indicator of antibiotic effectiveness, the minimum inhibitory concentration (MIC), which is the lowest concentration of NPs that inhibit the visible growth of the bacteria, was calculated using Prism9 software (version 2021) through the pre-established Lambert and Pearson method with nonlinear regression. Briefly, data with X were equal to the logarithm of the concentration and those with Y were proportional to the number of bacteria in the sample. Then, the data was fitted to a Gompertz model, hence fixing the bottom plateau, the span of the curve, the log of the inflection point and a slope factor. Finally, the MIC was defined from the slope [44].

#### 2.4.3. In Vitro Cytotoxicity Studies

For the in vitro cytotoxicity tests, primary human dermal fibroblasts (HDF) (Lonza, CC-2509, AMP, Hopkinton, MA, USA) and melanoma (MEL) cells (ATCC CRL-1619, Manassas, VA, USA) were cultured at 37 °C and 5% CO_2_ in a humidified atmosphere in Dulbecco’s Modified Eagle Medium (DMEM; Thermo Fisher Scientific, Waltham, MA, USA), supplemented with 10% fetal bovine serum (FBS; ATCC 30-2020™, American Type Culture Collection, Manassas, VA, USA) and 1% penicillin/streptomycin (Thermo Fisher Scientific). When the cells were 70–90% confluent, they were trypsinized, neutralized with fresh media and seeded into 96-well tissue culture plates (Thermo Fisher Scientific). HDF and MEL cells were then seeded at a final concentration of 5 × 10^4^ cells per well in 100 µL of cell medium after they were counted. The seeded well plates were kept in a humidified atmosphere at 37 °C and 5% CO_2_.

For the cytotoxicity assay, (3-(4,5-Dimethylthiazol-2-yl)-5-(3-carboxymethoxyphenyl)-2-(4-sulfophenyl)-2H tetrazolium) (MTS) (CellTiter 96® Aqueous One Solution Cell Proliferation Assay, Promega, Madison, WI, USA) was used to determine cellular metabolic activity, hence correlating with cell viability. After being seeded, cells were incubated for a period of 24 h at 37 °C in a humidified incubator with 5% CO_2_. Then, the culture medium was replaced with 100 µL of fresh cell medium containing various concentrations of the different BMNPs. Importantly, prior to in vitro assessment, BMNPs solutions were sterilized through UV exposure for 30 min inside a biohood.

Cells were cultured for an extra 24 h in the same conditions, followed by washing the cells with PBS and replacing the medium with 100 µL of the MTS solution (prepared using a mixing ratio of 1:5 of MTS:medium). After the addition of the solution, the 96-well plate was incubated for 4 h to allow for a color change. Then, the absorbance was measured at 490 nm on an absorbance plate reader (SpectraMAX M3, Molecular Devices, San Jose, CA, USA) for cell viability after exposure to the four different BMNPs and their respective concentrations. Cell viability was calculated by dividing the average absorbance obtained for each sample by the one obtained for the control sample and then multiplied by 100. Controls containing either cells and media or just media were also included. Cell experiments were carried out three times, accounting for a total of 12 data points that were organized in 24 h and 72 h experiments. Prior to in vitro assessment, BMNPs solutions were sterilized through UV exposure for 30 min inside a biohood.

#### 2.4.4. In Vitro Mechanistic Studies

To elucidate the mechanism of NPs cell death associated with both antibacterial and cytotoxic effects, ROS was quantified. Briefly, 2′,7′-dichlorodihydrofluorescein diacetate (H2DCFDA) was added to the mammalian cells that were seeded in a 96-well plate at a concentration of 5 × 10^4^ cells/mL in DMEM medium, along with different concentrations of BMNPs. The cells were cultured under standard culture conditions (37 °C in a humidified incubator with a 5% CO_2_ atmosphere) for 24 h before the experiment. The ROS indicator was reconstituted in anhydrous dimethyl sulfoxide (DMSO) to make a concentrated stock solution that was kept and sealed. The cell growth medium was then carefully removed, and a fixed volume of the indicator in PBS was added to each one of the wells at a final concentration of 10 μM. The cells were incubated for 30 min at an optimal temperature, and the loading buffer was removed thereafter. Fresh medium was added, and the cells were allowed to recover for a short time. Furthermore, positive controls were established with hydrogen peroxide (H_2_O_2_) to a final concentration of 50 μM and the baseline for the fluorescence intensity was quantified by subtracting the values of the controls. The fluorescence intensity was then observed by flow cytometry. Measurements were taken at a fluorescence wavelength of 530 nm when the sample was excited at 485 nm.

#### 2.4.5. Statistical Analysis

All experiments were repeated in triplicate (*N* = 3) and statistical significance was assessed using Student’s *t*-tests, with an alpha value less than 0.05 being statistically significant. Results were displayed as the mean ± standard deviation (Prism 9 software).

## 3. Results and Discussion

### 3.1. Synthesis and Optical Properties of Colloidal Bimetallic Ag/Au-NPs

**Figure 1:**
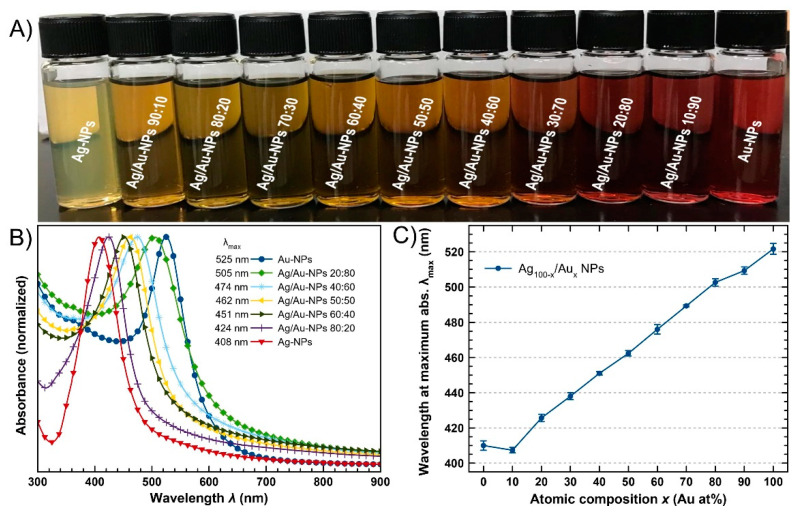
Optical properties of the colloidal Ag/Au-NPs. (**A**) Photograph of the colloids. From left to right: Ag-NPs to Au-NPs with Ag/Au-NPs having intermediate compositions. (**B**) Normalized absorption spectra of representative samples. (**C**) Wavelength at maximum absorption showing the average and standard deviation values from three independent sets of samples for each atomic composition.

Bimetallic Ag/Au-NPs was synthesized via a green wet-chemical method with a starch-mediated simultaneous reduction of metallic ions in an aqueous solution (Ag^+^(aq) and Au^3+^ in AuCl_4_^−^ (aq)). As the synthesis progressed, it was observed that the color of the aqueous solutions turned from colorless (in the case of Ag) or light yellow (in the case of Au) to a range of color from a yellowish (AgNPs) to brownish (Ag/Au-NPs) to reddish (AuNPs) clear colloid depending on the amount of the metal salt precursors added to each reaction mixture (Table S1). At the end of the reaction time, set at 3 h, the colorful colloids indicated the effective synthesis of the nanoparticles (Figure 1A).

Optical properties were studied by UV-vis spectroscopy. All of the colloidal metal nanoparticles exhibited a single absorption band corresponding to the coherent oscillation of the free electrons of quasi-spherical NPs excited by an incident electromagnetic wave with energy in the visible range. This optical phenomenon is known as the localized surface plasmon resonance (LSPR) in the metal nanostructures [45]. In each bimetallic nanosystem, the position of the wavelength at maximum absorption (λ_max_) depends on the atomic composition of the nanomaterials, i.e., for pure Ag-NPs, λ_max_ is localized at 408 nm, whereas for pure Au-NPs, the LSPR band is observed at around 525 nm, and for bimetallic Ag/Au-NPs, the absorption bands can be found between these two values (Figure 1B and Figure S1). Moreover, the fact that a single LSPR band without the presence of any other band or shoulder was observed in each spectrum is a strong indication that bimetallic nanoparticles with an alloy structure were produced [46,47,48]. This assumption was corroborated by EDX mapping conducted on the individual Ag/Au-NPs (vide infra).

To confirm the reproducibility of the synthesis method, Ag/Au-NPs were synthesized 3 separate times and analyzed by UV-vis spectroscopy each time. In Figure 1C, the position of λ_max_ was plotted as a function of atomic composition. It can be clearly observed that the standard deviation for λ_max_ was +/− 3 nm which highlights the strong reproducibility of the synthesis method.

In Figure S2, the change in the absorption spectra from selected colloidal BMNPs per time (up to 43 days) is depicted, in which a slight red-shift and a decrease in the absolute absorbance was observed. These changes may be related to an increase in the apparent average particle size due to agglomeration [49,50]. However, it can be concluded that the colloids were relatively stable within the first 40 days after synthesis. Starch is a biopolymer composed of polymeric chains, amylose and amylopectin, which contains a large amount of hydroxyl groups (reducing groups) [51] not only capable of reducing metallic ions to zero-valent metals, but also acts as an effective NP coating to control and stabilize the particles, preventing significant aggregation over time [36,52].

In Figure S3, we compared the simulated and experimental absorption spectra of BMNPs formulated at different atomic compositions. The simulated absorption spectra are in good agreement with the measured spectra of the nanoparticles in suspension. Moreover, both the simulated and the measured spectra showed that the resonance wavelength (λ_max_) of the BMNPs increased (or decreased) linearly with Au (or Ag) atomic content (Figure S4), this is in agreement with previous studies [41,46,53]. Discrepancies in the resonance linewidth between the simulated and experimental spectra for the larger Ag containing NPs can be attributed to the inhomogeneity of particle shape as observed by TEM (*vide infra*), i.e., having a combination of spherical and elliptical particles.

### 3.2. Starch as a Capping Agent Characterized by FTIR Spectroscopy

**Figure 2:**
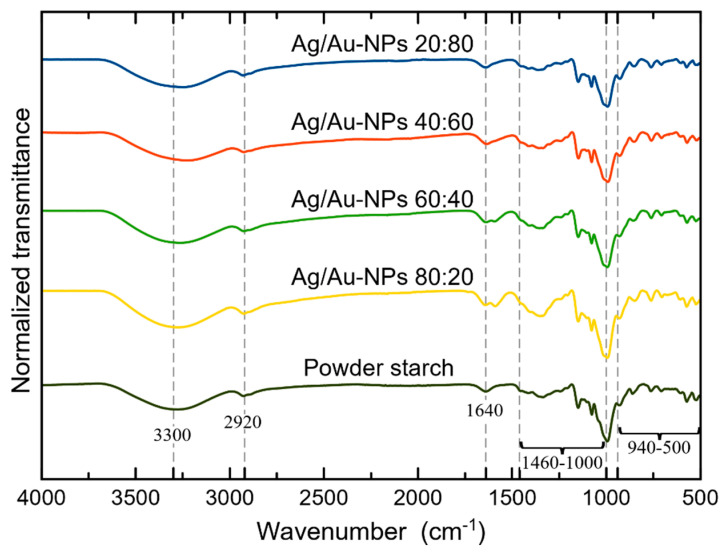
FTIR spectra of representative Ag/Au-NPs and powder starch as a reference.

FTIR spectroscopy measurements were done to determine the presence of the capping agent (starch) in the colloids. In Figure 2, the FTIR spectra of representative Ag/Au-NP samples and powder starch, used as a reference, are depicted. In general, the absorption bands observed in all of the BMNPs coincided with the typical functional groups in starch, i.e., a broad band around 3300 cm^−1^ attributed to the stretching of the hydroxyl group (–OH) and the bands at 2920 and 1640 cm^−1^ assigned to the antisymmetric stretching vibration of the C–H bond and the presence of H_2_O molecules in starch, respectively. The group of signals at 1000–1460 cm^−1^ correspond to glycosidic linkages and the region at 500–940 cm^−1^ is related to the glucose pyranose ring [54,55].

### 3.3. Structural Characterization Using XRD

**Figure 3:**
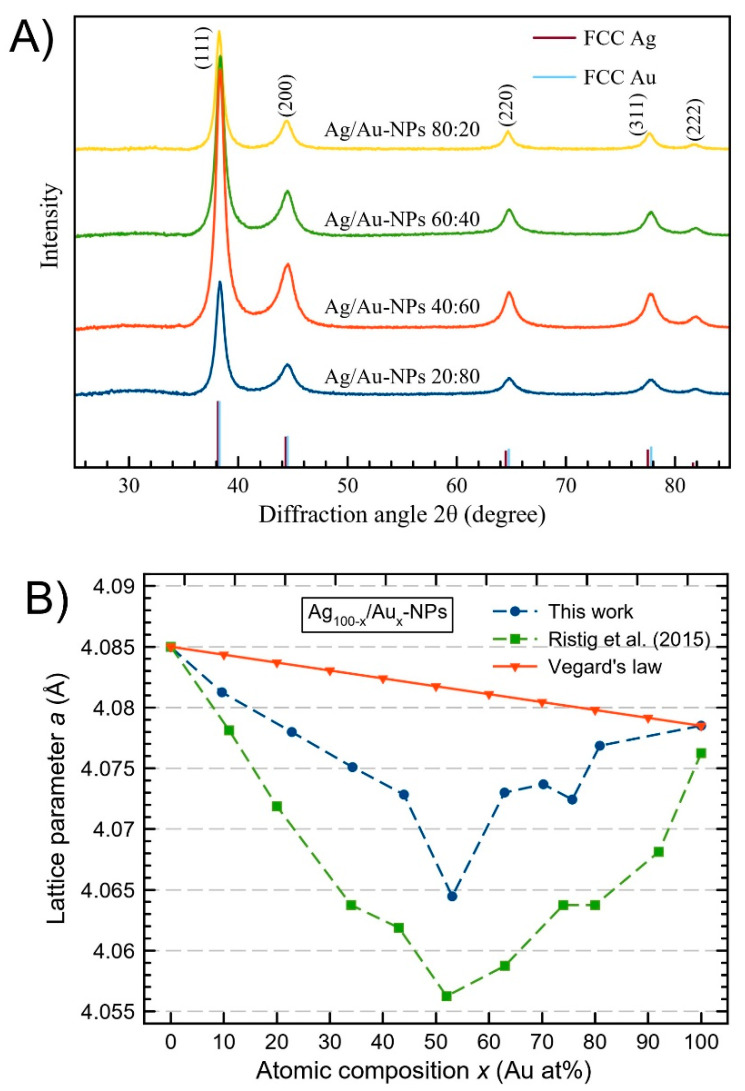
(**A**) XRD patterns of representative Ag/Au-NPs. (**B**) Lattice parameter a as a function of atomic composition (determined by ICP-MS) for the Ag/Au-NPs synthesized in this work compared with literature data [48] and the corresponding calculated values using Vegard´s law.

Powder X-ray diffraction (XRD) patterns of representative Ag/Au-NP samples are depicted in Figure 3A. In all of the experimental diffractograms, the diffraction peaks observed at 2θ ≈ 38.3, 44.4, 64.7, 77.7 and 81.8° corresponded to (111), (200), (220), (311) and (222), in agreement with FCC Ag and Au [56]. Bragg’s law was used to calculate the cubic lattice parameter (*a*) of the nanosystems [57]. The calculated values for the lattice parameter *a* for both pure Ag- and Au-NPs were 4.0850 and 4.0785 Å, respectively, and the bimetallic Ag/Au-NPs for all of the composition range had values close to these extreme samples (the complete set of XRD patterns can be found in Figure S5). These values were compared with the corresponding values calculated using Vegard´s law, which is a linear approximation for binary alloy systems to estimate the lattice parameter *a* from the alloy chemical composition through the equation *a*_Ag(100−*x*)_/_Au*x*_ = (100 − *x*)*a*_Ag_ + *xa*_Au_, where *a*_Ag_ and *a*_Au_ are the experimental lattice parameter of pure constituents and *x* is the nominal atomic composition of the alloy [58]. The atomic radius of Ag (1.445 Å) is a little bit bigger than the atomic radius of Au (1.442 Å) [58], however, when the Ag and Au atoms are added into the same lattice, electronic interactions between the outer electron shells of the Ag atoms and the surrounding Au atoms decrease the atomic radius of Ag atoms [59]. This phenomenon may explain why most of the Ag/Au-NPs synthesized in this work, understood as the alloy nanostructures, have smaller lattice parameters than the corresponding pure metals. In Figure 3B, a negative deviation with respect to the Vegard´s law for all of the concentration ranges for Ag/Au-NPs can be observed. A maximum negative deviation can be observed for the Ag/Au-NPs at 50:50 (around −0.017 Å) which agrees with the minimum experimental lattice parameter reported by Ristig et al. for Ag/Au-NPs [48]. In Table S2, the crystallite size (*L*), calculated using the Debye-Scherrer equation [60], is reported for the Ag/Au-NPs in the entire range of compositions. The calculated values for *L* are relatively close to the particle sizes obtained by TEM measurements (*vide infra*).

### 3.4. Particle Size and Morphology Characterization by STEM Analysis

**Figure 4:**
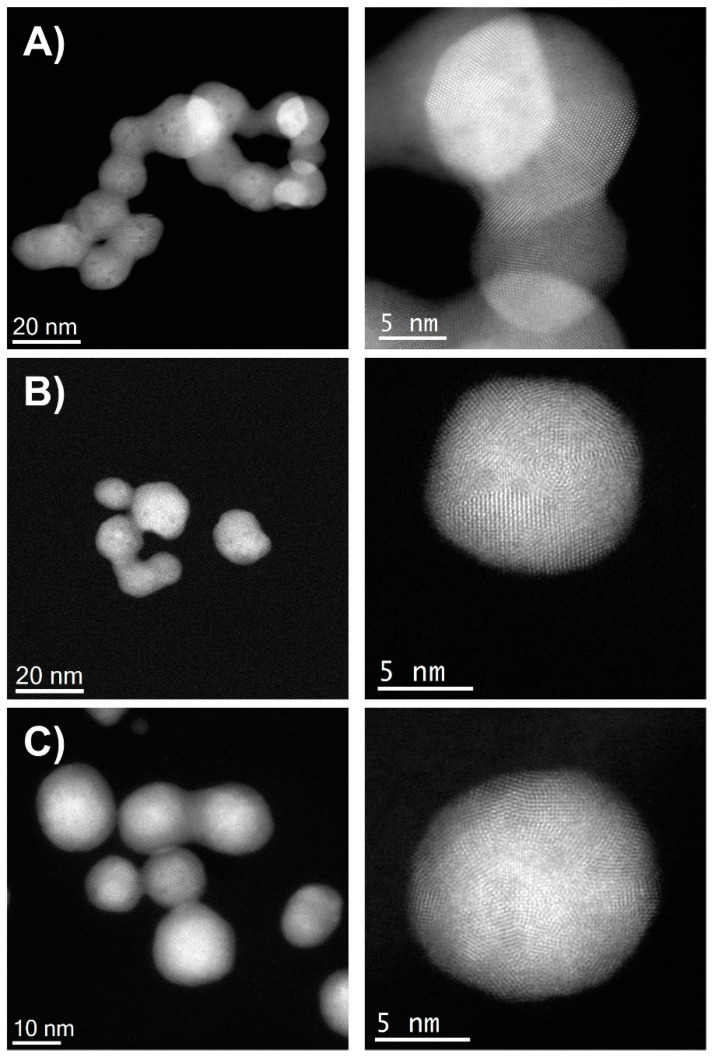
Cs-corrected STEM-HAADF analysis of BMNPs with Ag:Au ratios of (**A**) 20:80, (**B**) 40:60 and (**C**) 80:20. The left column shows low-magnification images while the right column shows atomic resolution of each respective sample.

Low-magnification Cs-corrected STEM-HAADF of representative BMNPs are presented in Figure 4. The left column depicts several BMNPs with a quasi-spherical shape and with mean diameters of 15.4 ± 2.2, 13.4 ± 2.9 and 14.3 ± 2.1 nm for Ag/Au-NPs with nominal atomic compositions of 20, 40 and 80 Ag atomic (%), respectively. In the case of the BMNP with an Ag:Au ratio of 20:80, it can be observed that a chain-like structure was formed. However, its corresponding absorption spectrum (Figure 1B) showed a single LSPR band which rules out the formation of an anisotropic nanostructure. Closer inspection of the structure (right column) revealed the formation of multi-twinned NPs regardless of the composition. Interestingly, a significant variation contrast was observed for the Ag rich material (Ag:Au ratio of 80:20). There was a significant variation contrast between the core and the outer part of the NPs, suggesting the formation of a core-shell structure (with a Au rich core and a Ag rich shell). Nevertheless, no Ag oxidation was observed on the surface, proving the positive effect of the starch on protecting these NPs.

### 3.5. Elemental Chemical Composition Analysis by ICP-MS, XPS and EDX

**Figure 5:**
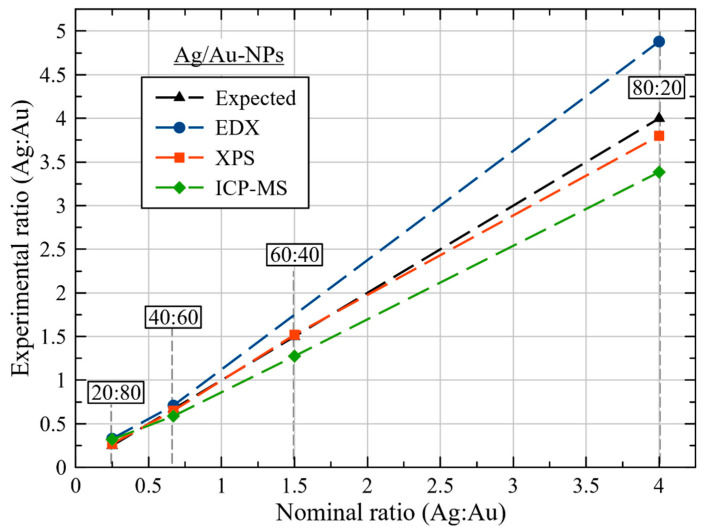
Chemical composition of Ag/Au-NPs with atomic ratios (Ag:Au) of 20:80, 40:60, 60:40 and 80:20 as determined by ICP-MS, EDX and XPS.

ICP-MS analysis was carried out to determine the chemical composition of the colloidal Ag/Au-NP materials (Figure S6). XPS and EDX have also been used to obtain independent composition measurements. In Figure 5, the relationship between the nominal and experimental composition with different techniques of selected Ag/Au-NP samples is compared. As can be observed, the metal compositions of the BMNPs are in good agreement with the composition of the initial feed metal precursors (nominal composition), meaning that the simultaneous co-reduction of the cations (Ag^+^ and Au^3+^ in AuCl_4_^−^) by starch was effective at the experimental parameters.

**Figure 6:**
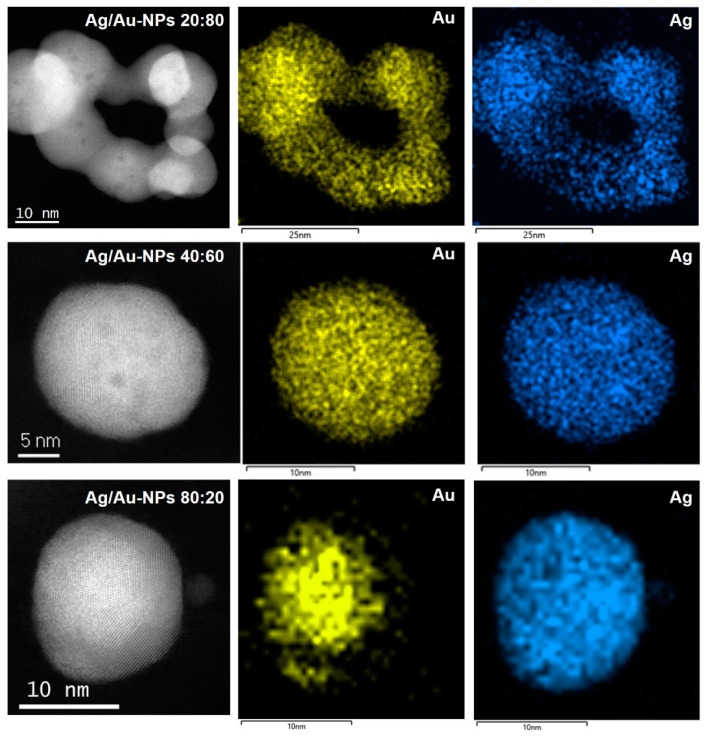
EDX mapping of individual Ag/Au-NPs with different atomic ratios (Ag:Au). 20:80 (**upper** panel); 40:60 (**middle** panel); and 80:20 (**lower** panel).

A more extensive chemical elemental analysis was achieved by EDX mapping on individual Ag/Au-NPs (Figure 6). In all cases, chemical maps were obtained for Ag and Au, as no other element was identified, besides C and O (from starch and Cu for the grid used). For the Ag/Au-NPs with atomic ratios (Ag:Au) of 20:80 and 40:60, a relative homogeneous composition was present in each sample, i.e., bimetallic alloy nanostructures were formed, which is in accordance with the UV-vis spectroscopic measurements (Figure 1B). In the case of the Ag/Au-NPs with a nominal composition of 80:20 (Ag:Au), a core-shell type structure (with a core rich in Au and a shell rich in Ag) was identified. Interestingly, the absorption spectrum from this sample (Figure 1B) showed the presence of only one band without bumps. In addition, the spectra from which the chemical maps were extracted are depicted in Figure S7, and the corresponding atomic ratio (Ag:Au) is plotted in Figure 5. It can also be noted that the experimental values are close to the ideal compositions, like the experimental compositions found by ICP-MS analysis.

**Figure 7:**
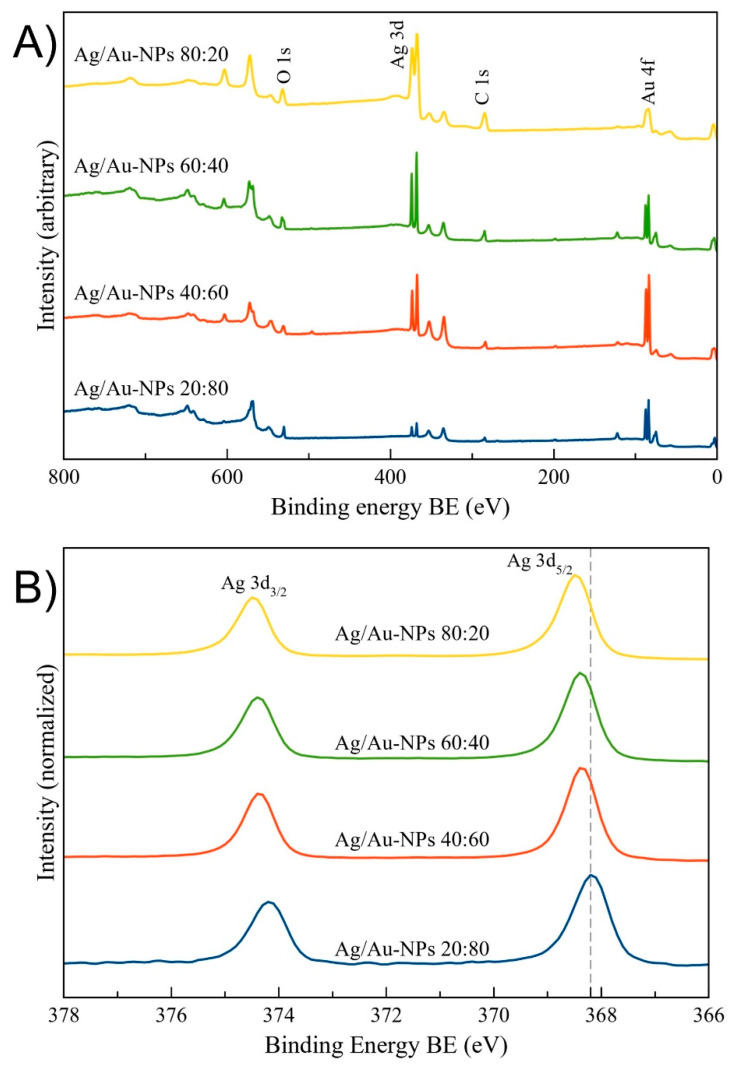
XPS characterization of Ag/Au-NPs with Ag:Au ratios of 20:80, 40:60, 60:40 and 80:20. (**A**) Wide energy range scans. (**B**) Ag 3d core level spectra. The dotted line corresponds to the BE of Ag 3d_5/2_ (368.2 eV) found in bulk Ag [66].

XPS analysis was performed on the 20, 40, 60 and 80 Ag at% Ag/Au-NPs. Figure 7A shows the wide energy range scan spectra of the samples, where Ag, Au, C and O were detected. A detailed analysis of the Ag 3d and Au 4f core level peaks (Figure 7B and Figure S8, respectively) showed the relative position of the corresponding bands with respect to bulk Ag (Ag 3d_5/2_ at a binding energy (BE) of 368.1 eV) [61] and Au (Au 4f_7/2_ at a BE of 84.0 eV) [62], respectively. For the case of the Ag 3d core level, there was a clear shift towards a higher BE as a function of increasing Ag content (Table S3), in accordance with Martínez et al. [63]. This shift is due to the primary effect of Au, where a depletion occurs in the d band accompanied by a nearly matching increase in s conduction electron character at the Au site [64]. Regarding the Au 4f core level, for all of the samples, the observed BE were all positively shifted with respect to the bulk value. With the exception of the Ag/Au-NPs at 60:40, there was a correlation in the BE shifts as a function of Au content (Table S3), i.e., at lower Au content, a higher positive BE shift was observed, in agreement with reported results [63,65]. The chemical composition of the BMNPs was determined by analyzing the integrated intensities of Ag 3d and Au 4f core level peaks. Moreover, the experimental atomic ratios (Ag:Au) (Table S4) agreed with the ICP-MS and EDX analyses, and they are also reported in Figure 5.

### 3.6. Antibacterial Properties of Ag/Au-NPs

To determine the antibacterial activity of four different Ag/Au-NPs (20:80, 40:60, 60:40 and 80:20), antibacterial assays (using colony forming units) were completed using two bacterial strains that show antibiotic resistance: methicillin-resistant *Staphylococcus aureus* (MRSA) and multidrug-resistant *Escherichia coli* (MDR-EC). Metal NP concentrations used for all of the experiments were between 5 and 20 µg/mL which were determined based on ICP-MS analysis. 

**Figure 8:**
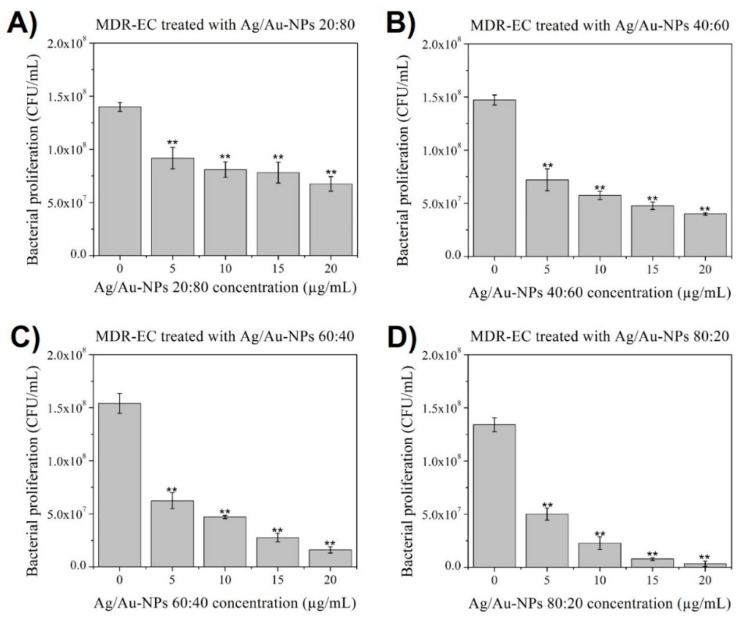
MDR-EC colony-counting assay after treatment for 8 h with Ag/Au-NPs at atomic ratios (Ag:Au) of (**A**) 20:80, (**B**) 40:60, (**C**) 60:40 and (**D**) 80:20. Data = mean ± SD, *N* = 3. ** *p* < 0.01 versus control (0 µg/mL concentration). The statistical analysis was based on a *t*-distribution.

For MDR-EC experiments (Figure 8), it was observed that the BMNPs with a larger amount of Au in their structure (20:80 and 40:60) led to a milder bacterial inhibition compared to those with a predominance of Ag in their composition (60:40 and 80:20). A clearer dose-dependent inhibition of bacterial growth was found when Ag/Au-NPs at 80:20 were added to the MDR-EC cultures, leading to a dramatical decay at medium and high NP concentrations (15 and 20 µg/mL, respectively). 

**Figure 9:**
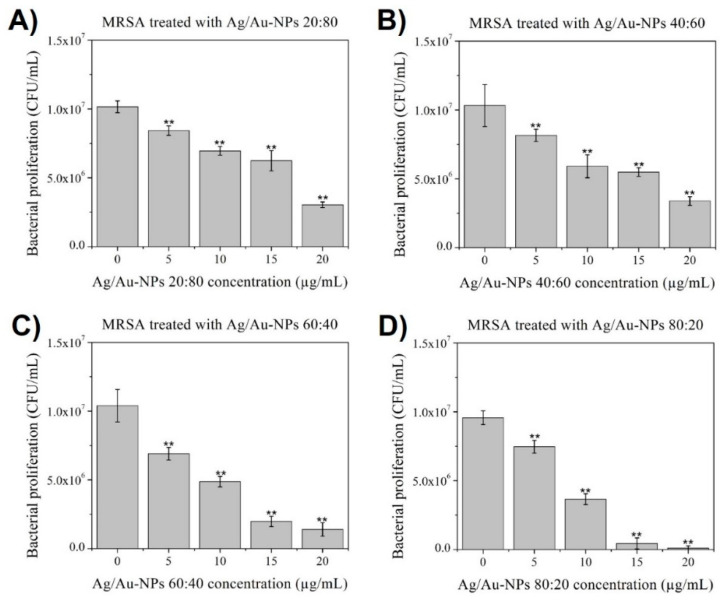
MRSA colony-counting assay after treatment for 8 h with Ag/Au-NPs with atomic ratios (Ag:Au) of (**A**) 20:80, (**B**) 40:60, (**C**) 60:40 and (**D**) 80:20. Data = mean ± SD, *N* = 3. ** *p* < 0.01 versus control (0 µg/mL concentration). The statistical analysis was based on a *t*-distribution.

When the Ag/Au-NPs were added to a MRSA culture (Figure 9), a similar behavior was found. Although there was statistical difference with the control, both Ag/Au-NPs at 20:80 and 40:60 displayed low inhibition compared to the control, while a dose-dependent behavior was clear for the Ag/Au-NPs at 60:40 which was more significant at 80:20, where medium and high doses (15 and 20 µg/mL, respectively) achieved a larger inhibition.

The results confirmed the antibacterial effect of the alloy-type BMNPs against drug-resistant pathogens, with a trend especially noticeable in the case of the NPs with a higher Ag content. Such a trend was recently observed for the antibacterial activity of Ag/Au-NPs alloys, prepared by a laser-based technique, against *Staphylococcus aureus* and *Escherichia coli*, concluding that the antibacterial effect of the NPs not only depends on the overall content of Ag in solution, but also relies on the composition of the nanoalloys [67]. In contrast, Padmos et al. did not find any noticeable trend in the antibacterial activity of Ag/Au-NPs at different atomic compositions against *Staphylococcus aureus* [68]. They proposed that the antibacterial activity of the BMNPs is a consequence of the location of the Ag atoms (i.e., their position on the surface or within the NP) and not the overall Ag concentration in the NP [68].

Moreover, the minimum inhibitory concentrations (MIC) for each of the tested bacterial strains were calculated and are shown in Table 1. As was expected, for both pathogens, the MIC values were lower for the rich Ag-content BMNPs. Despite the differences in composition, size and morphology of the NPs, as a comparison, the MIC values reported for carboxy methyl tamarind-coated Ag/Au-NPs against MDR-EC (ATCC BAA-2469™) was 6 ug/mL [69], which is slightly higher than the corresponding values found for the starch-capped Ag/Au-NPs with a composition of 60:40 and 80:20 (Ag:Au). In other work, the MIC value for Ag/Fe-NPs against MRSA was 250 µg/mL (or 250 ppm) [38].

### 3.7. Anticancer Activity of Ag/Au-NPs

**Figure 10:**
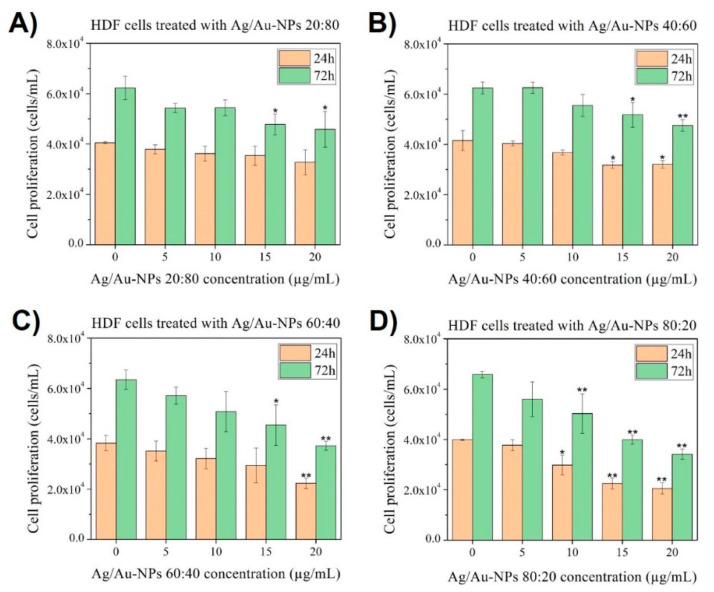
MTS assays on HDF cells in the presence of Ag/Au-NPs at atomic ratios (Ag:Au) of (**A**) 20:80, (**B**) 40:60, (**C**) 60:40 and (**D**) 80:20 at concentrations ranging from 5 to 20 µg/mL. Data = mean ± SD, *N* = 3. * *p* < 0.05 versus control (0 µg/mL concentration) and ** *p* < 0.01 versus control (0 µg/mL concentration). The statistical analysis was based on a *t*-distribution.

Cytotoxicity studies were conducted on human dermal fibroblasts (HDF) and melanoma cells to assess anticancer properties and cytocompatibility of the Ag/Au-NPs for over 24 and 72 h. When the BMNPs were added to HDF cells (Figure 10), three of the compositions analyzed (20:80, 40:60 and 60:40) were cytocompatible at low concentrations (5 and 10 µg/mL), whilst they became cytotoxic at medium and high concentrations (15 and 20 µg/mL, respectively). Finally, the Ag/Au-NPs at 80:20 were cytotoxic even at 10 µg/mL, which can be ascribed to the increased content of Ag within the nanostructures. A similar overall trend was observed in the literature when NIH-3T3 fibroblasts were treated with Ag/Au-NPs in which the cytocompatibility was higher when the Au content increased in the NP alloy [68].

**Figure 11:**
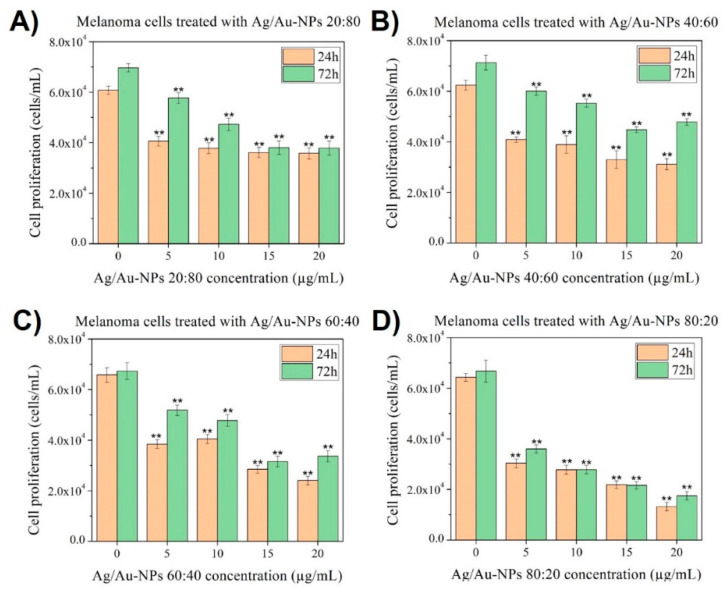
MTS assays on melanoma cells in the presence of Ag/Au-NPs with atomic ratios (Ag:Au) of (**A**) 20:80, (**B**) 40:60, (**C**) 60:40 and (**D**) 80:20 at concentrations ranging from 5 to 20 µg/mL. Data = mean ± SD, *N* = 3. ** *p* < 0.01 versus control (0 µg/mL concentration). The statistical analysis was based on a *t*-distribution.

On the other hand, when the same starch-capped BMNPs were added to melanoma cells (Figure 11), all of the compositions exhibited cytotoxic behavior against these cancer cells even at the lowest analyzed concentration, 5 µg/mL. Moreover, an important deviation from the normal growth of the controls was found, with medium and high concentrations (15 and 20 µg/mL, respectively) of both Ag/Au-NPs at 60:40 and 80:20 led to a significant anticancer pattern, with more than a 50% reduction in cell growth. Besides, the Ag/Au-NPs at 20:80 and 40:60, with a higher Au content, had a smaller anticancer effect, with a less clear dose-dependent inhibition of cell proliferation. The Ag-dependent cytotoxicity properties of Ag/Au-NPs agrees with the anticancer effect observed when BMNPs of different compositions were used against HCT116 and 4T cell lines (specifically, human colon carcinoma and breast cancer cell lines, respectively) [70].

**Figure 12:**
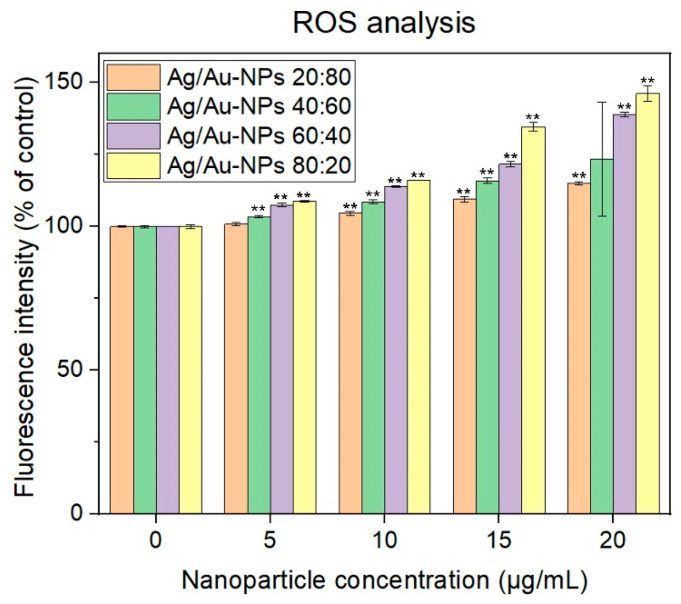
ROS analysis on melanoma cells in the presence of the different Ag/Au-NPs at concentrations ranging from 5 to 20 µg/mL. Data = mean ± SD, *N* = 3; ** *p* < 0.01 versus control (0 µg/mL concentration). Statistical analysis performed was based on a *t*-distribution.

To understand the mechanisms that led to such cell death, ROS analysis was performed on experiments with melanoma cells. As can be seen in Figure 12, an increased concentration of BMNPs led to a rise in ROS production within the media compared to the controls only containing melanoma cells. At the same time, a higher content of Ag within the BMNP structure led to a larger production of ROS. It has been reported in the literature that Ag nanoparticles induce the production of ROS and trigger apoptosis in a size-dependent manner—the smaller the nanoparticles, the more ROS generated [71]. Besides, AuNPs can induce ROS in a similar manner, but to a lesser extent than their Ag counterparts [72].

## 4. Conclusions

In this work, Ag/Au bimetallic nanoparticles were synthesized using an environmentally friendly, reproducible and simple method, in which the biopolymer starch was used as a reducing and capping agent. The synthesized nanoparticles showed a quasi-spherical morphology with particle sizes in the range of 10–15 nm. EDX mapping analysis revealed that Ag and Au atoms were homogeneously distributed in the NPs with atomic ratios Ag:Au of 20:80, 40:60 and 60:40, i.e., alloy-type nanostructures were formed, while a core-shell like structure was observed for the 80:20 sample. As a consequence, the optical properties of the colloids were linearly dependent on the atomic composition. Moreover, the potential biomedical applications of the Ag/Au bimetallic nanoparticles were tested against relevant antibiotic-resistant bacterial strains, showing that the NPs have a dose-dependent antimicrobial effect that was especially noticeable with an increase in Ag content in the nanosystems. Additionally, the BMNPs showed no or low cytotoxicity when exposed to human dermal fibroblasts at medium concentrations (up to 10 µg/mL for the Ag/Au-NPs with atomic ratios Ag:Au of 20:80, 40:60 and 60:40), while rendering an anticancer effect to melanoma cells that can be attributed to the overproduction of ROS in the media. Therefore, the presently fabricated bimetallic nanoparticle systems were shown to be a reliable, reproducible and effective source of nanomaterials with biomedical potential against multidrug-resistant pathogens and cancer cells.

## Figures and Tables

**Table 1:** MIC values found for colloidal Ag/Au-NPs against multidrug-resistant *Escherichia coli* (MDR-EC) and methicillin-resistant *Staphylococcus aureus* (MRSA).

Ag/Au-NPs Sample	MIC MDR-EC (µg/mL)	MIC MRSA (µg/mL)
20:80	20.8	18.1
40:60	18.5	16.3
60:40	5.3	10.2
80:20	4.5	7.9

## Data Availability

The data presented in this study are available upon reasonable request to the corresponding authors.

## Supplementary Materials

The following supporting information can be downloaded at: https://www.mdpi.com/article/10.3390/nano12050779/s1, Figure S1: Normalized absorption spectra of colloidal Ag/Au-NPs in the complete atomic chemical composition range with steps of 10 at%; Figure S2: Study of the stability over the time of Ag/Au-NPs; Figure S3: Comparisons between simulated and experimental absorption spectra for Ag/Au-NPs with different chemical compositions; Figure S4: Comparison between the LSPR band positions obtained in the 3D FDTD simulations and the experimental values; Figure S5: XRD spectra of the Ag/Au-NPs with different chemical compositions; Figure S6: Experimental chemical composition of the Ag/Au-NPs found by ICP-MS as a function of nominal composition; Figure S7: EDX spectra of Ag/Au-NPs; Figure S8: Au 4f core level peak for Ag/Au-NPs; Table S1: Experimental conditions for the synthesis of Ag/Au-NP colloids; Table S2: Crystallite size (*L*) Ag/Au-NPs extracted from XRD analysis; Table S3: Shift of the binding energy (ΔBE) for Ag 3d_5/2_ and Au 4f_7/2_ with respect to the bulk values of selected Ag/Au-NP samples; Table S4: Chemical composition of the selected Ag/Au-NP samples.
