# Supplementary material for: Composition-Dependent Cytotoxic and Antibacterial Activity of Biopolymer-Capped Ag/Au Bimetallic Nanoparticles against Melanoma and Multidrug-Resistant Pathogens
Source: Nanomaterials (Basel). 2022 Feb 25;12(5):779. doi: 10.3390/nano12050779 (PMC8912067; doi:10.3390/nano12050779)
Supplement: Supplementary file 1 [file nanomaterials-12-00779-s001.zip › nanomaterials-1585531-supplementary.pdf]

nanomaterials

Supplementary Materials

Composition-dependent cytotoxic and antibacterial activity of biopolymer-capped Ag/Au bimetallic nanoparticles against melanoma and multidrug-resistant pathogens

Alfonso Nieto-Argüello [1], David Medina-Cruz [2], Yeremi S. Pérez-Ramírez [1], Sergio A. Pérez-García [3], Miguel. A. Velasco-Soto [1], Zeinab Jafari [1], Israel De Leon [1], María Ujué González [4], Yves Huttel [5], Lidia Martínez [5], Álvaro Mayoral [6,7,8], Thomas J. Webster [2], José M. García-Martín [4] and Jorge L. Cholula-Díaz [1,*]

[1] School of Engineering and Sciences, Tecnologico de Monterrey, Eugenio Garza Sada 2501, Monterrey 64849, N.L., Mexico; alfonsonieto25@gmail.com (A.N.-A.); yeremi_said@hotmail.com (Y.S.P.-R.); miguel.velasco85@tec.mx (M.A.V.-S.); ZJafari@tec.mx (Z.J.); ideleon@tec.mx (I.D.L)
[2] Department of Chemical Engineering, Northeastern University, Boston, MA 02115, USA; davidmedina-crz@gmail.com (D.M.-C.); websterthomas02@gmail.com (T.J.W.)
[3] Centro de Investigación en Materiales Avanzados, S. C. (CIMAV), Unidad Monterrey, Alianza Norte 202, C.P. 66628, Apodaca, NL, Mexico; alfonso.perez@cimav.edu.mx
[4] Instituto de Micro y Nanotecnología, IMN-CNM, CSIC (CEI UAM+CSIC), Isaac Newton 8, Tres Cantos 28760, Spain; maria-ujue.gonzalez@csic.es (M.U.-G.); josemiguel.garcia.martin@csic.es (J.M.G.-M.)
[5] Instituto de Ciencia de Materiales de Madrid, ICMM-CSIC, Sor Juana Inés de la Cruz 3, Madrid 28049, Spain; huttel@icmm.csic.es (Y.H.); lidia.martinez@icmm.csic.es (L.M.)
[6] Instituto de Nanociencia y Materiales de Aragón (INMA), CSIC-Universidad de Zaragoza, Pedro Cerbuna, Zaragoza 50009, Spain; amayoral@unizar.es
[7] Laboratorio de Microscopías Avanzadas (LMA), Universidad de Zaragoza, Zaragoza 50018, Spain
[8] Center for High-Resolution Electron Microscopy (ChEM), School of Physical Science and Technology (SPST), ShanghaiTech University, 393 Middle Huaxia Road, Pudong, 201210, Shanghai, China
* Correspondence: jorgeluis.cholula@tec.mx

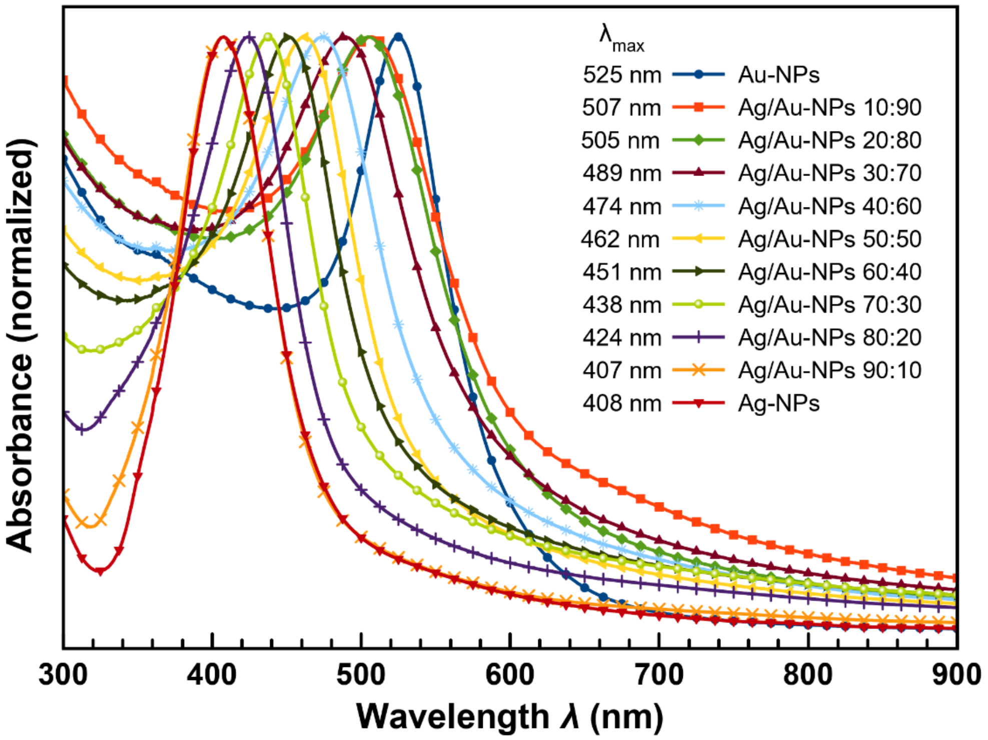

Figure S1. Normalized absorption spectra of colloidal Ag/Au-NPs in the complete atomic chemical composition range with steps of 10 at%.

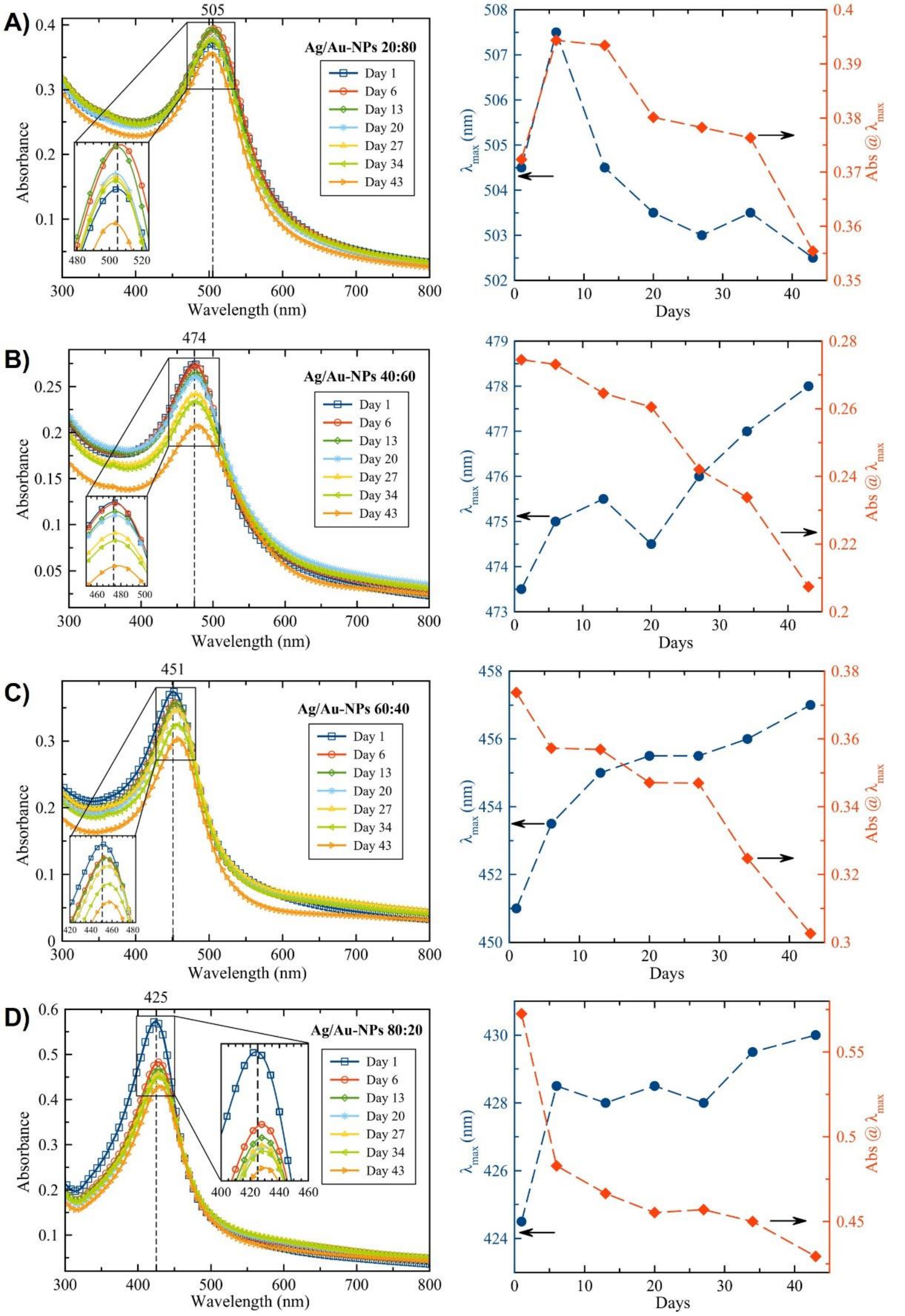

Figure S2. Study of the stability over the time of Ag/Au-NPs. Absorption spectra (left panels) and shift of λ_{max} and absorbance at the given λ_{max} as a function of time (right panels) for colloidal Ag/Au-NPs with Ag:Au ratio of **A**) 20:80, **B**) 40:60, **C**) 60:40, **D**) 80:20.

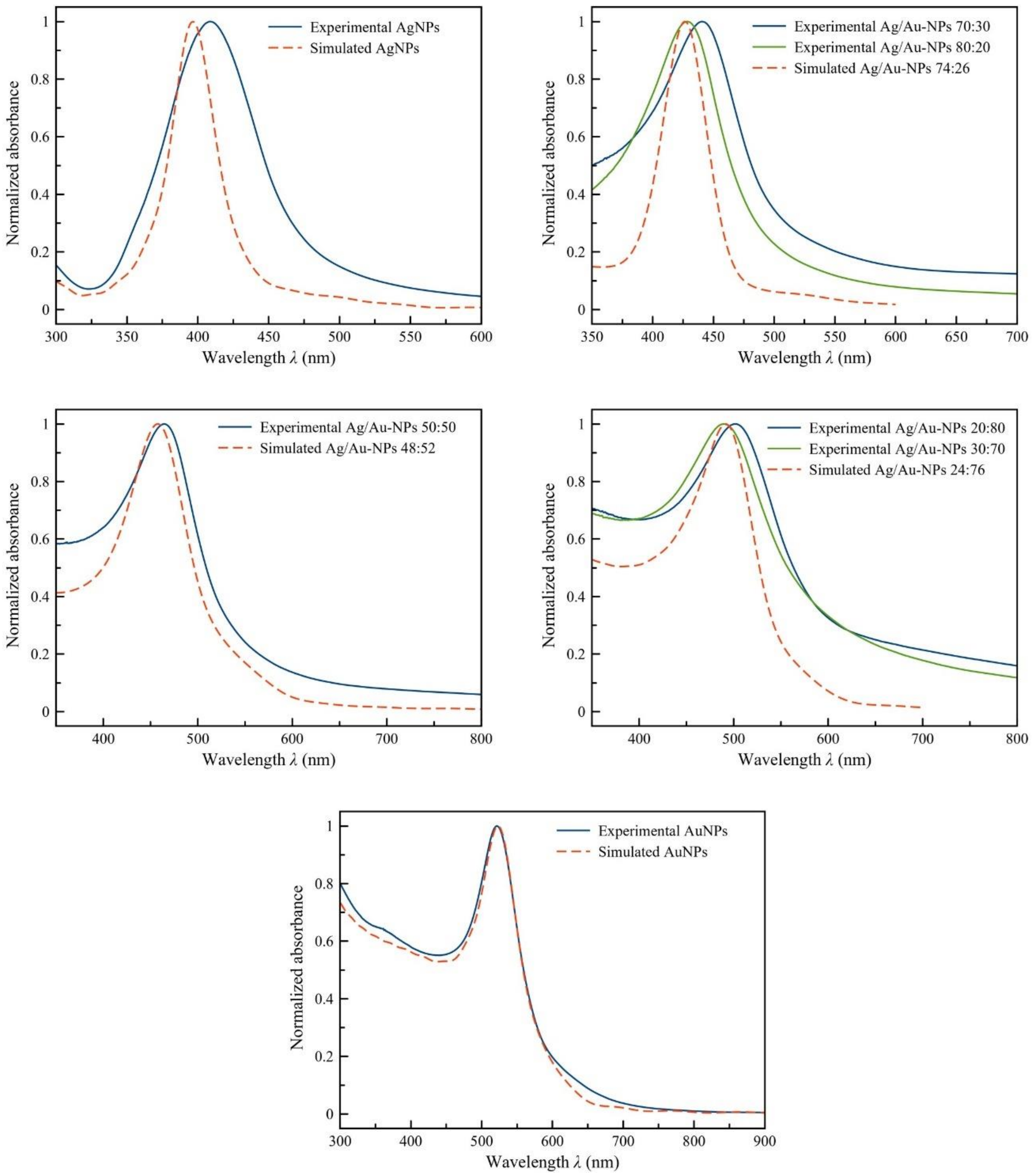

Figure S3. Comparisons between simulated and experimental absorption spectra for Ag/Au-NPs with different chemical compositions. The simulated spectra were calculated using 3D FDTD analysis.

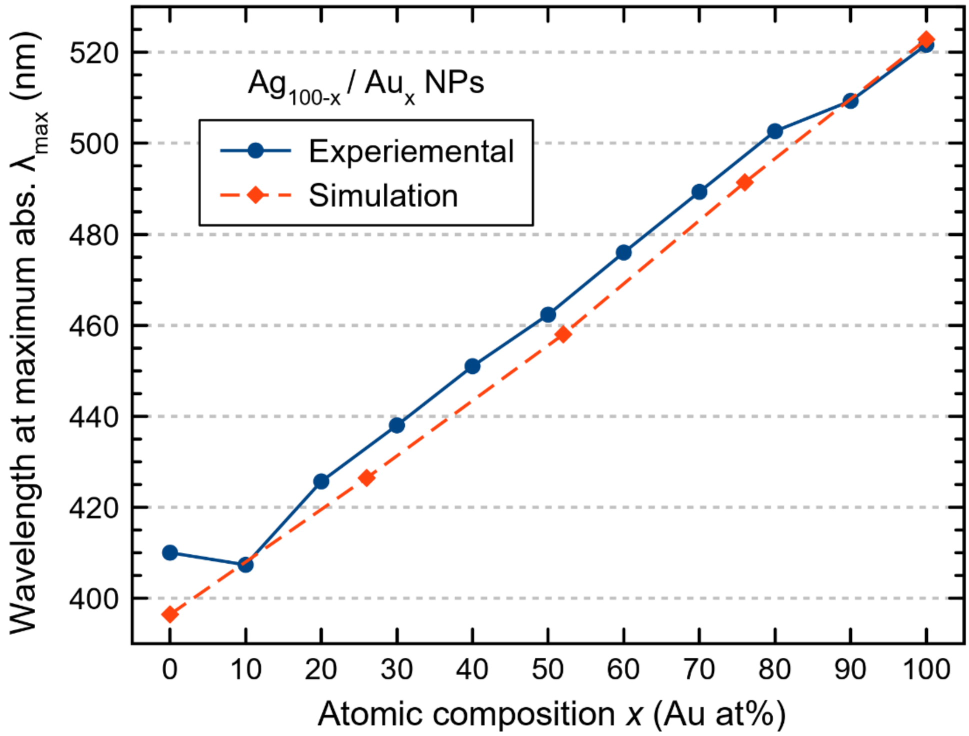

Figure S4. Comparison between the LSPR band positions obtained in the 3D FDTD simulations and the experimental values.

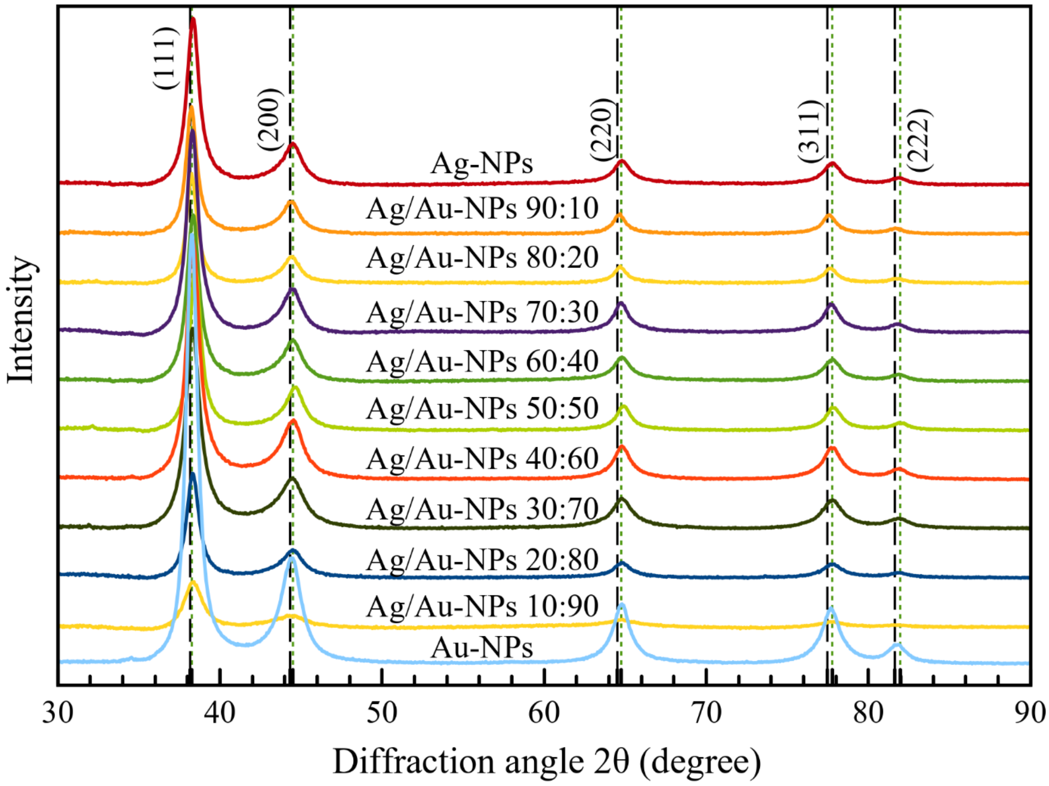

Figure S5. XRD spectra of the Ag/Au-NPs with different chemical compositions. Discontinuous and dotted lines correspond to expected diffraction peaks for FCC Ag and Au[1], respectively.

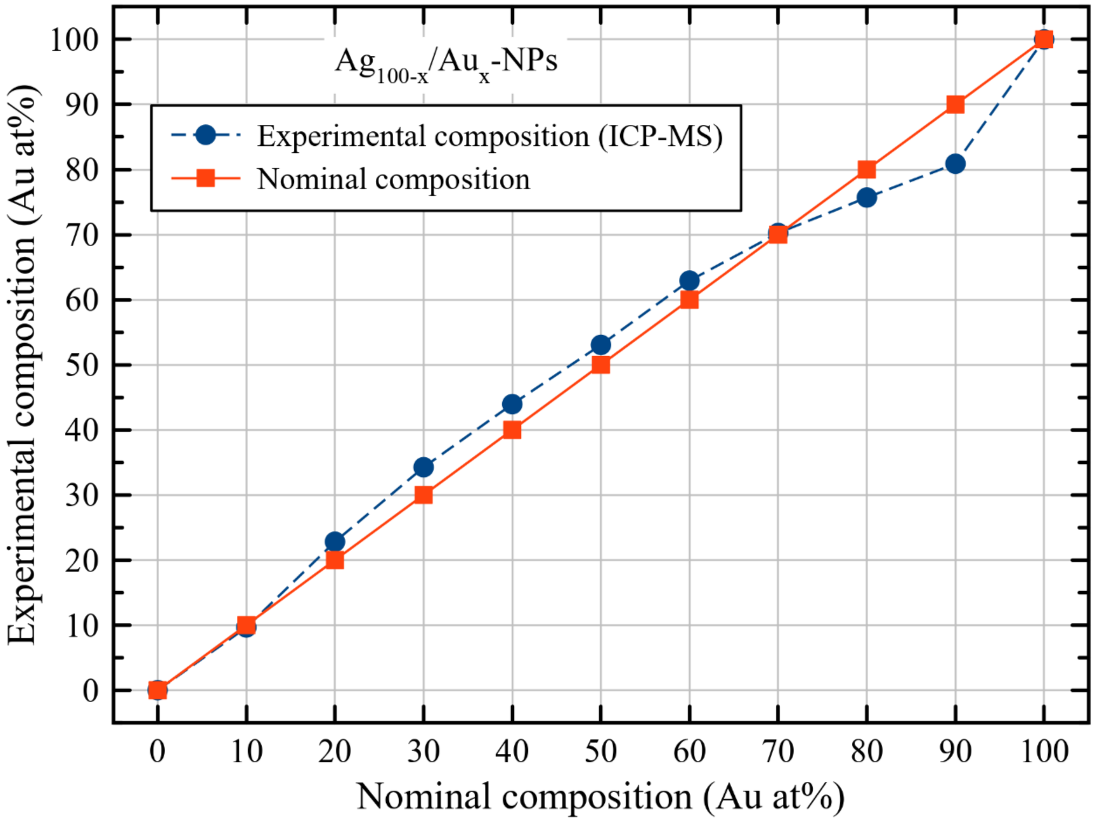

Figure S6. Experimental chemical composition (blue dots) of the Ag/Au-NPs found by ICP-MS as a function of nominal composition. The red squares correspond to the nominal composition of each sample. The blue dashed and red lines are guides for the eye.

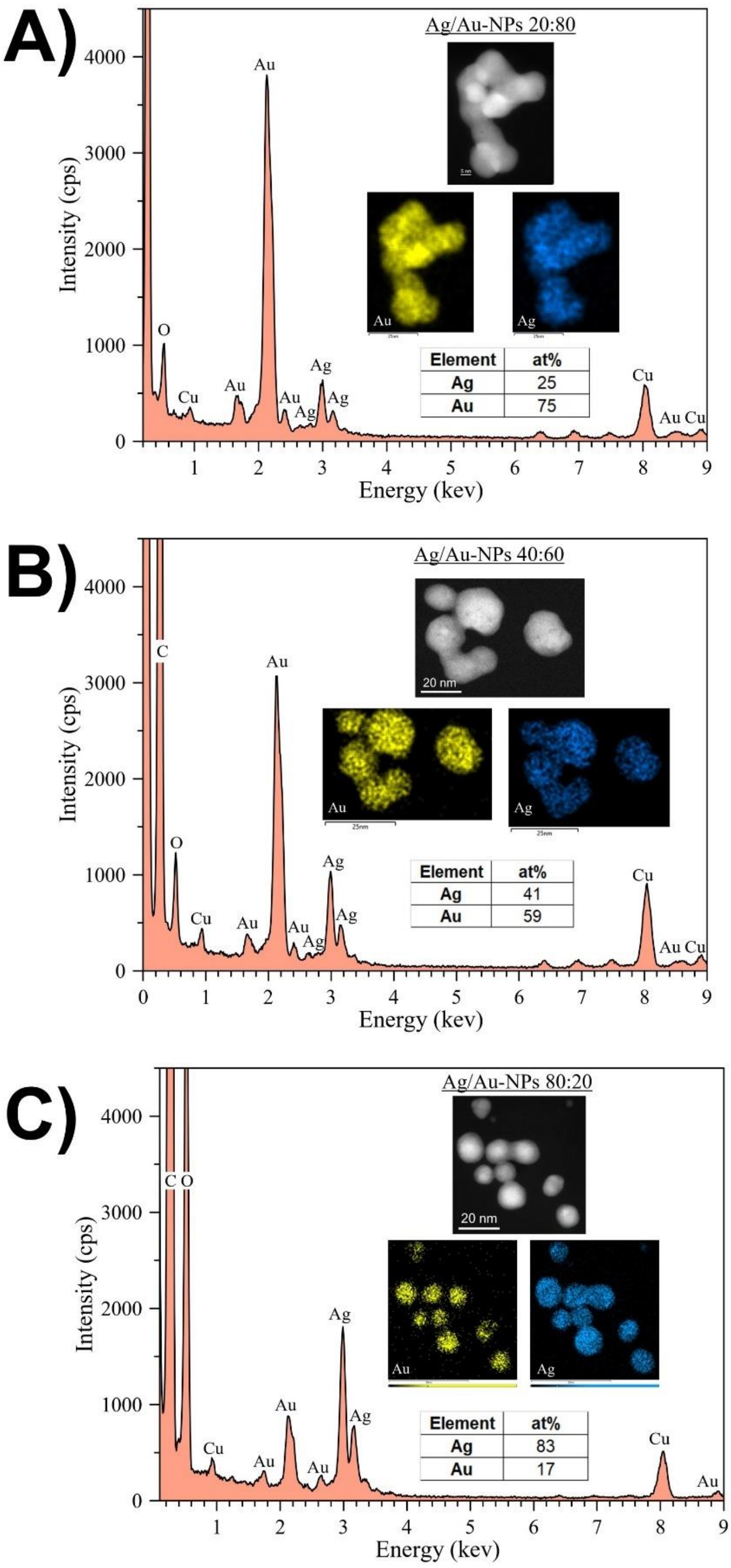

Figure S7. EDX spectra of Ag/Au-NPs with Ag:Au ratio of **A**) 20:80; **B**) 40:60; and **C**) 80:20.

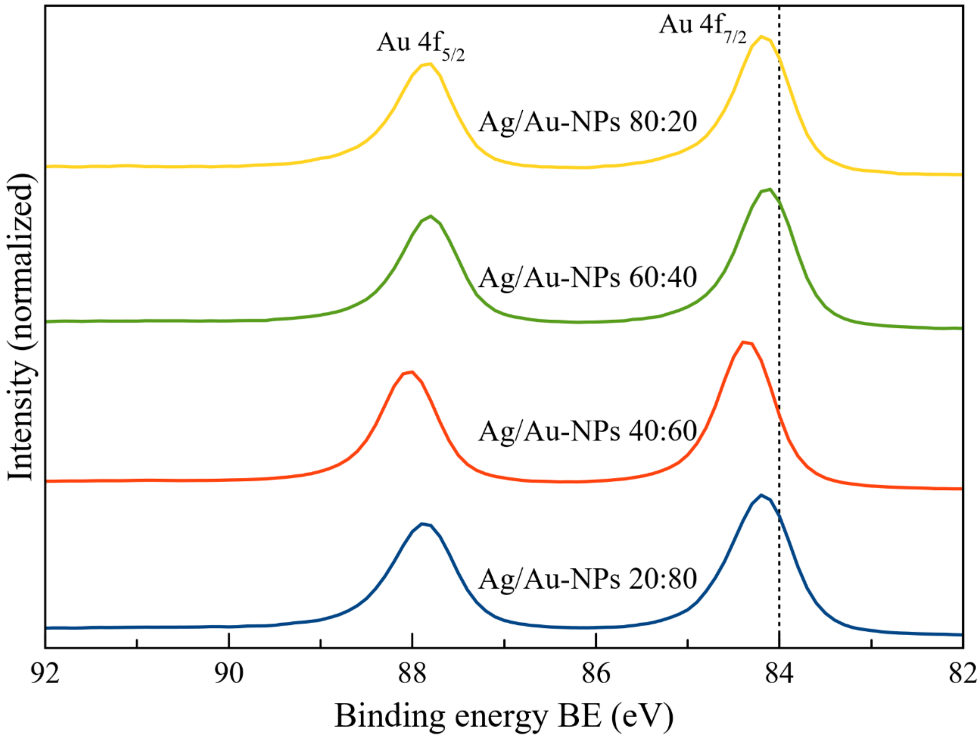

Figure S8. Au 4f core level peak for Ag/Au-NPs with Ag:Au ratio of 20:80, 40:60, 60:40 and 80:20. The dotted line correspond to the BE of Au $4f_{7/2}$ (84.0 eV) found in bulk Au[2].

Table S1. Experimental conditions for the synthesis of Ag/Au-NP colloids. In all samples, 500 μL of starch 1% (w/v) solution was used.

Atomic Ratio (Ag:Au)	$AgNO_3$ 25 mM Solution (μL)	$NaAuCl_4$ 25 mM Solution (μL)
100:0	250	0
90:10	450	50
80:20	400	100
70:30	350	150
60:40	300	200
50:50	250	250
40:60	200	300
30:70	150	350
20:80	100	400
10:90	50	450
0:100	0	250

Table S2. Crystallite size (*L*) Ag/Au-NPs extracted from XRD analysis (Figure S5).

Ag/Au-NP Sample	Crystallite Size (*L*, in nm)
100:0	15.3 ± 2.6
90:10	12.9 ± 3.9
80:20	13.1 ± 2.1
70:30	11.4 ± 1.7
60:40	9.6 ± 2.7
50:50	10.6 ± 2
40:60	8.7 ± 1.7
30:70	6.7 ± 1.3
20:80	8.3 ± 1.1
10:90	7.6 ± 2.8
0:100	11.2 ± 3.8

Table S3. Shift of the binding energy (ΔBE) for Ag $3d_{5/2}$ and Au $4f_{7/2}$ with respect to the bulk values (368.1 and 84.0 eV, respectively) of selected Ag/Au-NP samples obtained from the Ag 3d (Figure 7B) and Au 4f (Figure S8) core level peaks.

Ag/Au-NP Sample	Ag $3d_{5/2}$ Shift (eV)	Au $4f_{7/2}$ Shift (eV)
20:80	0.1	0.18
40:60	0.28	0.36
60:40	0.31	0.33
80:20	0.41	0.38

Table S4. Chemical composition of selected Ag/Au-NP samples extracted from the Ag3d (Figure 7B) and Au4f (Figure S8) core level peaks for Ag and Au, respectively.

Ag/Au-NP Sample	Expected Atomic Ratio Ag:Au	Experimental Atomic Ratio Ag:Au
20:80	0.25	0.27
40:60	0.67	0.65
60:40	1.50	1.52
80:20	4.00	3.80
